# Patient safety culture in the Operating Room of an emergency hospital in Amazonas: perspectives from the healthcare team

**DOI:** 10.1590/0100-6991e-20243743-en

**Published:** 2024-07-05

**Authors:** FRANCISCO DEYVIDY SILVA OLIVEIRA, RANNA ABADIAS PESSOA, CLEBER LOPES CAMPÊLO, LEONARDO PESSOA CAVALCANTE

**Affiliations:** 1 - Universidade Federal do Amazonas, Programa de Pós-graduação em Cirurgia - Manaus - AM - Brasil; 2 - Universidade Federal do Amazonas, Faculdade de Medicina - Manaus - AM - Brasil; 3 - Universidade do Estado do Amazonas - Manaus - AM - Brasil

**Keywords:** Patient Safety, Organizational Culture, Quality of Health Care, Cultura de Segurança, Segurança do Paciente, Cultura Organizacional, Qualidade da Assistência à Saúde

## Abstract

**Introduction:**

The concept of safe care permeates health institutions around the world, however, it is necessary to understand the safety culture of an institution to improve the provision of safety to patients and professionals.

**Methodology:**

Cross-sectional study with a quantitative approach. The sample was made up of 119 health professionals who made up the multidisciplinary team at the surgical center from August to September 2021, where data collection took place. The Hospital Survey on Patient Safety Culture (HSOPSC) instrument was used to evaluate the twelve dimensions that make up patient safety culture. Data analysis was carried out using descriptive statistics, to evaluate the reliability of the responses to the HSOPSC instrument, the Cronbachs Alpha test was used.

**Results:**

Of the twelve dimensions evaluated, there was no dimension considered strong for patient safety in the unit. The dimensions with potential for patient safety were “Expectations and actions of the supervisor/manager to promote patient safety”; “Teamwork within units” and “Organizational learning - continuous improvement”, while all other dimensions were evaluated as weak for patient safety. 39.50% of participants consider patient safety in the unit to be regular, despite this, 89.91% of participants reported not having made any event notifications in the last 12 months.

**Conclusion:**

The study highlighted the need to strengthen all dimensions of the patient safety culture by the team at the hospital studied, as none of them were identified as strong.

## RESUMO

**Introdução:**

O conceito do cuidado seguro permeia as instituições de saúde no mundo todo, no entanto, é preciso entender a cultura de segurança de uma instituição para melhorar a oferta de segurança aos pacientes e aos profissionais.

**Metodologia:**

Estudo transversal com abordagem quantitativa. A amostra foi composta por 119 profissionais de saúde que compunham a equipe multiprofissional do centro cirúrgico nos meses de agosto a setembro de 2021, onde ocorreu a coleta de dados. Foi utilizado o instrumento Hospital Survey on Patient Safety Culture (HSOPSC), para avaliar as doze dimensões que compõem a cultura de segurança do paciente. A análise dos dados foi feita através de estatística descritiva, para avaliar a confiabilidade das respostas ao instrumento HSOPSC, utilizou-se o teste Alpha de Cronbach.

**Resultados:**

Das doze dimensões avaliadas, não houve nenhuma dimensão considerada forte para segurança do paciente na unidade. As dimensões com potencial para segurança do paciente foram “Expectativas e ações do supervisor/chefia para a promoção da segurança do paciente”; “Trabalho em equipe dentro das unidades” e “Aprendizado organizacional - melhoria contínua”, enquanto todas as outras dimensões foram avaliadas como fracas para segurança do paciente. 39,50% dos participantes consideram a segurança do paciente na unidade como regular, apesar disso 89,91% dos participantes relataram não ter realizado nenhuma notificação de evento nos últimos 12 meses.

**Conclusão:**

O estudo apontou a necessidade do fortalecimento de todas as dimensões da cultura de segurança do paciente pela equipe do hospital pesquisado, uma vez que nenhuma delas foi apontada como forte.

## INTRODUCTION

Patient safety is defined as the reduction of unnecessary harm associated with health care to an acceptable minimum of the risk^1^. It is a current theme, with wide dissemination worldwide, since it is directly linked to the quality of health care, as well as pointing out to organizational and structural characteristics of the institution^2^.

Despite years of efforts and important advances since the publication of the report “To err is human” in 1999, a milestone for the visibility of patient safety in the world, progress has been slower than expected. The health system continues to operate with low reliability, still causing many unnecessary harms to patients, especially in developing countries^3^.

Brazil is considered one of the countries with the highest frequency of avoidable adverse events worldwide^4^. One study showed that the incidence of adverse events in Brazilian hospitals was 7.6% and the proportion of preventable events was 66.7%^5^. Concerned about this scenario, in 2013 the Ministry of Health launched the National Patient Safety Program (PNSP), which highlights the creation of a patient safety culture (PSC) within health institutions as one of the first pillars of patient safety^6^.

PSC is a product of group and individual values, attitudes, perceptions, and competencies, which determine a pattern of behavior and commitment to the institution’s safety management^7^.

The main obstacle to the achievement of safe care is health organizations weakened safety culture, reinforcing negative attitudes, hindering the promotion of high-quality, effective, efficient, and profitable care, thus being unable to offer a safe work environment^8^.

Errors and adverse events occur in all care settings, including Operating Rooms, which are considered high-risk sectors. In the Operating Room, complex, precise, multi-technology, multidisciplinary activities are developed, with a strong dependence on individual performance, but with a great need for teamwork, often marked by stress and pressure^9^.

Understanding PSC enables a view directed to the institution’s real problems and needs, reducing the occurrence of avoidable adverse events, unnecessary expenses resulting from care failures, and patient insecurity, promoting improvements in the quality of provided care^10^.

Thus, evaluating PSC contributes to the recognition of the organizational situation, signals the opportunities for improvement that exist in the unit, with the aim of boosting quality in care and, consequently, patient safety, in addition to being useful in monitoring the interventions implemented, as an evaluation method^11^. 

In view of this scenario, the objective of this study was to evaluate the patient safety culture from the perspective of the multidisciplinary team of the Operating Room (OR) in a hospital in the state of Amazonas, Northern Brazil.

## METHODS

This is a cross-sectional study with a quantitative approach. The study was carried out in the OR of a reference hospital in urgency and emergency of the state health network, located in the city of Manaus, Amazonas, Brazil, from August to September 2021.

The study population was composed of the entire OR multidisciplinary staff, consisting of assistant physicians/surgeons, nurses, nursing technicians, nursing assistants, radiology technicians, pharmacy technicians, and administrative assistants. Sampling was non-probabilistic for convenience.

As an inclusion criterion, the research subjects should have a minimum weekly workload of 20 hours. We excluded professionals who were on sick leave, maternity leave, or on leave for other reasons during data collection. We also excluded participants who answered less than one entire section of the instrument or answered less than half of the items of the entire instrument in different sections, and those who had the same response in all items.

During the operationalization of the research, we approached all professionals individually, when they were available, and instructed them about the objectives of the research and the completion of the instrument. With the professionals who, for some reason, were not available to respond on the date of the approach, the researchers made attempts on three subsequent dates.

To respond to the research objectives, we used the Hospital Survey on Patient Safety Culture (HSOPSC) questionnaire, created by the Agency for Healthcare Research and Quality (AHRQ) in 2004^12^, already translated and validated for the Portuguese language in Brazil^13^.

The HSOPSC is composed of 53 items built on the Likert Scale format, graded into five points that reflect the degree of agreement, ranging from 1 (strongly disagree or never) to 5 (strongly agree or always). The degree of patient safety was measured by the instrument on a different scale from the other items, and was evaluated by a 5-point scale, ranging from 1 (very poor) to 5 (excellent), and the number of safety events reported in the last 12 months was measured by responses ranging from “no notification” to “21 or more notifications^13^”. Of the items in the instrument, 42 assess PSC, one is open to comments on patient safety, and the remaining items refer to information related to the characterization of the participants.

The analysis of this instrument allows us to measure the 12 dimensions that compose PSC, namely: D1: Teamwork within the units; D2: Expectations and actions of the supervisor/manager to promote patient safety; D3: Organizational learning - continuous improvement; D4: Hospital management support for patient safety; D5: General perception of patient safety; D6: Return of information and communication about errors; D7: Openness to communication; D8: Frequency of reported events; D9: Teamwork among hospital units; D10: Adequacy of professionals; D11: Internal transfers and shift change; D12: Non-punitive response to error.

We entered the data generated by the HSOPSC into a Microsoft^®^ Excel^®^ spreadsheet and analyzed them using the statistical software R and RStudio^14^. For the descriptive analysis of the variables, we calculated the proportion and absolute frequency. We measured the Cronbach’s Alpha to assess the reliability and consistency of the data produced by the instrument. For this test, values above 0.70 are considered acceptable, and the closer to 1, the more reliable the test is^15^.

The evaluation of the safety culture was based on the percentage of neutral, positive, and negative responses obtained in each dimension on PSC. “Strong areas of patient safety” in the hospital were defined as those whose positively written items obtained 75% of positive responses (“strongly agree” or “agree”), or those whose negatively written items obtained 75% of negative responses (“strongly disagree” or “disagree”). Similarly, those whose items obtained 50% or less of positive responses were considered “fragile areas of patient safety” and in need of improvement. Results between 50% and less than 75% were considered potential areas of patient safety^12^.

This study met all the recommendation criteria of Resolution 466/2012 of the National Health Council16. It was appraised and approved by the Ethics in Research Committee (CEP) of the Federal University of Amazonas, with CAAE number 48131821.2.0000.5020 and opinion number 4.903.352.

## RESULTS

A total of 119 professionals from the multidisciplinary team participated in this study. Most were female professionals, 73 (61.35%), with a predominance in the age group of 31 to 35 years (46.22%), and who had direct contact with patients (96.64%). Regarding the professional category, nursing technicians were the ones who most filled out the questionnaires, 53 (44.53%), followed by 44 (36.97%) assistant physicians/surgeons. 

As for professional experience, most had between one and five years of work (56.30%), with a weekly workload of 20 to 39 hours per week (63.02%). Sociodemographic characteristics are shown in Table 1.

**Table 1:** Sociodemographic characteristics of participants. Manaus - AM, Brazil, 2021.

Variable	Total	%
Sex*		
Female	73	61.35
Male	43	38.65
Age group* (years)		
<25	09	7.56
25-30	10	8.40
31-35	55	46.22
36-40	14	11.78
41-45	13	10.92
46-50	14	11.76
>50	04	3.36
Length of time working in current specialty or profession* (years)		
1-5	67	56.30
6-10	19	16.00
11-15	11	9.24
16-20	16	13.44
>20	06	5.02
Weekly workload (hours)		
20-39	75	63.02
40-59	24	20.18
Variable	Total	%
≥ 60	20	16.80
Job Title/Function		
Physician Assistant/Surgeon	44	36.97
Nurse	15	12.60
Nursing technician	53	44.53
Nursing Assistant	03	2.52
Technician (Radiology, pharmacy)	02	1.68
Administrative Assistant	02	1.68
Direct contact with patients*		
Yes	115	96.64
No	04	3.36

Source: Researcher, based on the HSOPSC questionnaire. Manaus - AM, Brazil, 2021.

Table 2 shows the results of the Cronbach’s Alpha index, which ranged from 0.43 to 0.88. The “Frequency of reported events” dimension obtained the highest reliability index, and the “Adequacy of professionals” dimension, the lowest. However, when evaluating the overall instrument reliability, we obtained 0.70, indicating good reliability of the HSOPSC in this study.

**Table 2:** Cronbach’s Alpha from HSOPSC dimensions in the operating room. Manaus - AM, Brazil, 2021.

Dimension	Alpha
D1: Teamwork within the units	0.6049
D2: Expectations and actions of the supervisor/manager to promote patient safety	0.5029
D3: Organizational learning - continuous improvement	0.5180
D4: Hospital management support for patient safety	0.6393
D5: General perception of patient safety	0.5006
D6: Return of information and communication about errors	0.7693
D7: Openness to communication	0.5034
D8: Frequency of reported events	0.8833
D9: Teamwork among units	0.5975
D10: Adequacy of professionals	0.4395
D11: Internal transfers and shift change	0.4577
D12: Non-punitive response to error	0.4539

Table 3 shows the percentage of negative, neutral, and positive responses for each of the 12 PSC dimensions. We found no dimension that could be pointed out as a strong area for PSC. The dimensions with the greatest potential for patient safety were “Expectations and actions of the supervisor/manager for the promotion of patient safety”; Organizational learning - continuous improvement”, and “Teamwork within units”.

**Table 3:** Negative, neutral, and positive responses of the 12 dimensions of the translated version of the OR HSOPSC.

Dimensions (*)	(%) Negative	(%) Neutral	(%) Positive
D1	23.53	22.27	54.20
D2	18.27	23.73	58.00
D3	24.37	20.73	54.90
D4	34.45	29.70	35.85
D5	40.76	21.00	38.24
D6	35.30	26.05	38.65
D7	31.93	22.70	45.37
D8	43.70	19.05	37.25
D9	35.50	33.82	30.68
D10	44.75	20.37	34.88
D11	42.65	27.95	29.40
D12	64.70	18.50	16.80

The other dimensions were classified as fragile for patient safety. The dimensions with the lowest percentage and which are configured as areas that need to advance in patient safety were “Non-punitive responses to errors” and “Internal transfers and shift change”.

**Figure 1:**
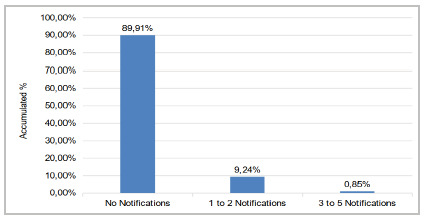
Distribution of responses to the item “In the last 12 months, how many event notifications have you filled?” in the OR.

Figure 1 shows the number of adverse events reported in the last 12 months prior to the survey. Most professionals (89.91%) did not report any adverse event, 9.24% reported one to two events, and one professional (0.85%) reported three to five.

**Figure 2:**
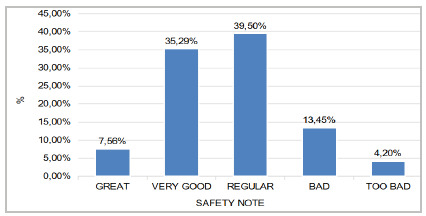
Distribution of responses to the item “Please assess patient safety in your area/unit of work in the hospital” in the OR.

Figure 2 shows the degree of patient safety. We observed that 39.50% evaluated patient safety as regular and only 7.56% as excellent.

## DISCUSSION

Through the application of the HSOPSC questionnaire, it was possible to evaluate the patient safety culture in the Operating Room of a state emergency referral hospital in Northern Brazil.

Although the HSOPSC instrument presented good overall reliability, we did not obtain the same results regarding the reliability of the dimensions separately. This result may be related to the population characteristics and the variability of the responses. 

We could not identify any strong areas in this study. Most of them were evaluated as fragile, indicating that the PSC is not established in the studied unit.

In this study, the dimensions “Expectations and actions of the supervisor/manager for the promotion of patient safety” and “Organizational learning - continuous improvement” were classified according to the criteria established by AHRQ as potential for PSC. These dimensions are directly related to the management for patient safety improvement. 

The dimension “Teamwork within the units” was another that presented a potential culture. The level of collaboration among these team members has a positive impact on the quality of health service delivery, and that the lack of harmony among professionals in a unit is one of the main barriers to achieving safe care^17^.

Of the 12 dimensions evaluated, nine of them were classified as fragile. The dimension with the highest percentage of fragility in the unit was “Non-punitive responses to errors”. The professionals believe that there is a culture of guilt and punishment in the unit and that reporting the mistake would harm them in some way. 

The fragility of this dimension is consistent with the high number of professionals who did not notify any event in the last twelve months. Efforts are needed to change this scenario, since, as previously pointed out, the professionals in this OR see the error as a learning opportunity.

The dimension “Internal transfers and shift change” brings the understanding that the unit’s professionals perceive that information about patients is not passed on effectively. 

Establishing strategies to strengthen communication, with specific information from the patient, is essential for the success of this process. The recommended quality tool for the units in this process of improving communication during shift change is the Situation Background Assessment Recommendation (SBAR), adopted internationally in health services, enabling the development of critical thinking and consolidating communication skills^19^.

When comparing the dimension “Teamwork between hospital units” and the dimension “Teamwork within the unit”, we noticed that professionals who work in the same sector develop a cooperative work, different from when they work with professionals from other sectors.

Professionals who perform their activities in an individual and non-interrelated way generate an environment of competition that is linked to a weak organizational culture, which is usually centralized and rigid, not providing an interdisciplinary work atmosphere^24^.

The dimension “Adequacy of professionals” was also evaluated as weak. The professionals in the study OR believe that the workloads are excessive and that the working hours and the number of professionals in the unit are insufficient to provide quality care aimed at patient safety.

Work overload is pointed out as one of the main causes of the occurrence of adverse events. A relationship between the size of the staff and the in-hospital mortality was carried out and pointed to a reduction of up to 7% in the mortality rate in environments with correct dimensioning^25^.

Another dimension identified as fragile was “Hospital management support for patient safety”. The result indicates that the employees of the hospital’s OR, despite having a good relationship with their supervisors, do not perceive their efforts to strengthen patient safety, inferring little commitment from the upper management.

Having senior management committed to patient safety, including adequate structural conditions, adequate materials and equipment, and sufficient personnel favors PSC and makes process planning more effective^26^.

The dimension “Frequency of reported events” presented the lowest rate of positive responses. The unit’s professionals do not usually report adverse events, making it impossible to create strategies for patient-centered care. 

Professionals need to be aware of the occurrence of errors and receive feedback on the changes that are implemented, as the strengthening of communication and the creation of bonds of trust between professionals are important properties of PSC^22^. When evaluating the dimension “Return of information and communication about errors”, we once again perceived effective communication as a limiting factor for PSC.

The evaluation of the dimension “Openness to communication” shows that there is resistance to dialogue about errors on the part of professionals, probably due to the focus centered on the author of the error. Identifying weaknesses in error reporting can provide important input for improvement.

When comparing this result with those obtained in the “Non-punitive response to error” dimension, we infer that, with the presence of weaknesses in these dimensions, the patient’s safety measure may be deficient and the exposed events may be minimized. 

The survey also pointed out that most participants did not make any notification in the prior 12 months. This fact may be associated with underreporting, causing damage to the entire system and not necessarily pointing to the reality of the frequency of events in the institution. PSC recommends that events be reported to allow the analysis of the causes and enable preventive education measures^23^.

When evaluating the dimension “General perception of patient safety”, we noticed that professionals believe that the tools and processes of the unit and the actions that are being used for patient safety are not being adequate or are not sufficient to prevent the occurrence of errors.

Even though the institution does not have strong areas for patient safety and also reinforces areas that are critical, most of the professionals who participated in the research classified patient safety in the unit as regular, demonstrating a low perception of the real scenario of the institution in which they work. 

As a limiting factor of the study, we point out the time taken to answer the questionnaire, due to the length of the instrument, considering that the research subjects were approached in their work environment, which is a place full of unforeseen events. The lack of knowledge about the basic concepts of patient safety may also have been a limitation.

As a strong point of this research, it was carried out with the entire multidisciplinary team of the Operating Room of the studied institution and not with only one professional category, as observed in other studies, contributing to support managerial decision-making, prioritizing the improvement of patient safety in the unit.

## CONCLUSION

This research made it possible to evaluate the patient safety culture in the Operating Room of a state hospital in Amazonas, where we could identify any strong area for patient safety.

Despite the high rate of dimensions classified as fragile areas, professionals perceive patient safety as regular. There was a high number of professionals within the OR who do not report adverse events.

The results indicate that the institution needs a systematized thinking in favor of the safety culture in the Operating Room, considering the human condition subject to errors and, for this, it needs to invest in changing this culture, where managers must support safe practices that help the development of a positive culture.
